# Deep Eutectic Solvents-Based Ultrasonic-Assisted Dispersive Liquid–Liquid Microextraction for the Determination of Organophosphorus Pesticides in Honeysuckle Dew Samples

**DOI:** 10.3390/molecules29143423

**Published:** 2024-07-21

**Authors:** Kangmiao Guo, Xiaokun Wu, Fan Zhang, Ying Cao, Zenglei Tan, Shuwen Xiao, Lijie Wu

**Affiliations:** 1 College of Chinese Materia Medica, Tianjin University of Traditional Chinese Medicine, 10 Poyanghu Road, West Area, Tuanbo New Town, Jinghai District, Tianjin 301617, China; 15383693621@163.com (K.G.); 18338595752@163.com (F.Z.); five43c@163.com (Y.C.); 15971623433@163.com (Z.T.); 15900241255@163.com (S.X.); 2 Department of Neurology, North China University of Science and Technology Affiliated Hospital, Tangshan 063000, China; wu_xkun@126.com

**Keywords:** deep eutectic solvents, ultrasound-assisted extraction, organophosphorus pesticides, honeysuckle dew samples, dispersion liquid–liquid microextraction

## Abstract

A deep eutectic solvent (DES) with the ability to change from hydrophilic to hydrophobic was designed and synthesized and applied to the determination of organophosphorus (OPP) pesticides in honeysuckle dew samples. Choline chloride, phenol, and tetrahydrofuran (THF) were used as the hydrogen bond acceptor, hydrogen bond donor, and demulsifier, respectively. Eight OPP pesticides were extracted by DES coupled with ultrasonic-assisted extraction (UA) and then chromatographed by GC-MS. DES used as an extract solvent has the advantages of high extraction efficiency, low cost, and environmental protection. Furthermore, DES is compatible with GC-MS. The single factor experiment design and Box–Behnken design (BBD) were applied to the optimization of experimental factors, including the type and composition of extraction solvent, type of demulsifier solvent, the volume of DES and THF, pH of sample solution, and ultrasonic time. Under the optimum experimental conditions, the high degree of linearity from 0.1 to 20.0 ng mL^−1^ (R^2^ ≥ 0.9989), the limits of detection from 0.014 to 0.051 ng mL^−1^ (S/N = 3), and the recoveries of analytes from 81.4 to 104.4% with relative standard deviation below 8.6%. In addition, the adsorption mechanism of OPPs on DES was explored by adsorption kinetic studies. These results have demonstrated that the present method has offered an effective, accurate, and sensitive methodology for OPP pesticides in honeysuckle dew samples, and this method provides a reference for the detection of pesticide residues in traditional Chinese medicine.

## 1. Introduction

Lonicerae Japonicae Flos (LJF) (*Lonicera japonica* Thun.) as a traditional Chinese medicine with a main property of clearing heat and eliminating toxins has been used widely in the treatment of diseases; it can cure wind-heat common cold and hot blood poison dysentery [1]. There are many kinds of Chinese patent medicines based on LJF, such as honeysuckle dew, Yinhuang granules, Shuanghuanglian oral liquid, Qingkailing oral liquid, etc. Among them, honeysuckle dew is widely used. Honeysuckle dew is an over-the-counter Chinese patent medicine mainly composed of LJF. The function of honeysuckle dew is mainly to clear away heat and detoxify heat. It is suitable for sunstroke, miliaria rash, furuncle, and so on, which affect the lung and stomach and are caused by summer heat. When a large number of heat rashes appear due to summer heat and humidity in children, honeysuckle dew can also be used to wipe the skin to relieve symptoms. Thus, honeysuckle dew is widely used in life [2].

However, the problem of insect pests is serious in the planting of LJF, and the use of organophosphorus (OPP) insecticides is more common. In recent years, a scandal of pesticide residues in honeysuckle has been reported from time to time [3,4,5]. OPP pesticides are mostly phosphoric acid or phosphoric acid derivatives containing phosphoryl (P=O) or thiophosphoryl (P=S). It has become one of the most widely used pesticides in the world due to its broad spectrum, high efficiency, and fast degradation [6,7]. Studies have pointed out that long-term exposure to low-dose OPPs can lead to neurological conduction dysfunction and irreversible damage to the nervous system [8]. With the increasing demand for LJF, the food and drug safety risks caused by pesticide residues are receiving more and more attention. Therefore, it is necessary to establish a simple, rapid, efficient, and green method for the detection of OPP pesticides in Chinese patent medicines containing LJF.

The traditional methods for the separation and enrichment of OPPs are solid phase extraction (SPE), liquid–liquid extraction (LLE), solid phase microextraction (SPME), and liquid phase microextraction (LPME). However, these methods are time-consuming and cumbersome. Dispersed liquid–liquid microextraction (DLLME) was proposed by Ahmadi in 2006. It is a miniature liquid–liquid extraction form which uses a micro-liter volume of extraction solvent. It has the obvious advantages of a high preconcentration coefficient, rapid operation, simple operation, high extraction efficiency, and low sample requirements. It has been widely used in the determination of toxic and harmful substances in food and environmental samples [9,10,11].

In order to conform to the concept of “green chemistry”, deep eutectic solvents (DESs) were first proposed by Abbott et al. [12]. A ‘deep eutectic solvent’ is a mixture of pure compounds for which the eutectic point temperature is below that of an ideal liquid mixture [13]. DESs are defined as homogeneous eutectic mixtures obtained by mixing two or more pure components (liquids or solids, ions or neutral molecules) acting as a hydrogen bond acceptor (HBA) and hydrogen bond donor (HBD) [14]. The properties of DESs used as an extraction solvent in LPME methods provide significant advantages. DESs have good solubility and can dissolve inorganic substances such as gas, metal ions, and a variety of organic substances insoluble in water. It is widely used in sample pretreatment [15]. In addition, DESs have the characteristics of biocompatibility, recyclability, biodegradability, and low or non-toxicity [16]. It is a kind of analog of ionic liquid and is one of the five green solvents recognized by scientists today. It has been successfully used in DLLME as extraction solvents for the preconcentration and separation of organic pollutants such as methadone [17], heavy metals [18], endocrine disrupting chemicals [19], pesticides [20], aromatic amines [21], oxyprenylated phenylpropanoids [22], and warfarin [23] in environmental samples, food, and biological samples. Compared with conventional extraction methods, DES coupled with DLLME could obtain more advantages [24]. However, there are no reports about the extraction of pesticide residues from traditional Chinese medicine such as honeysuckle dew samples.

In this study, a rapid, effective, and eco-friendly DES-UA-DLLME method for the separation and preconcentration of eight OPPs, including diazinon, tolclofos-methyl, pirimiphos-methly, phosalone, malathion, fenthion, fenamiphos, and bolster in honeysuckle dew samples prior to GC-MS determination, was developed. This technique combines extraction and preconcentration of the analytes into one step, simplifies the analytical step, and saves operation time. The developed method was successfully applied to the simultaneous determination of trace levels of OPPs in real samples.

## 2. Results and Discussion

### 2.1. Characterization of DES

**Figure 1:**
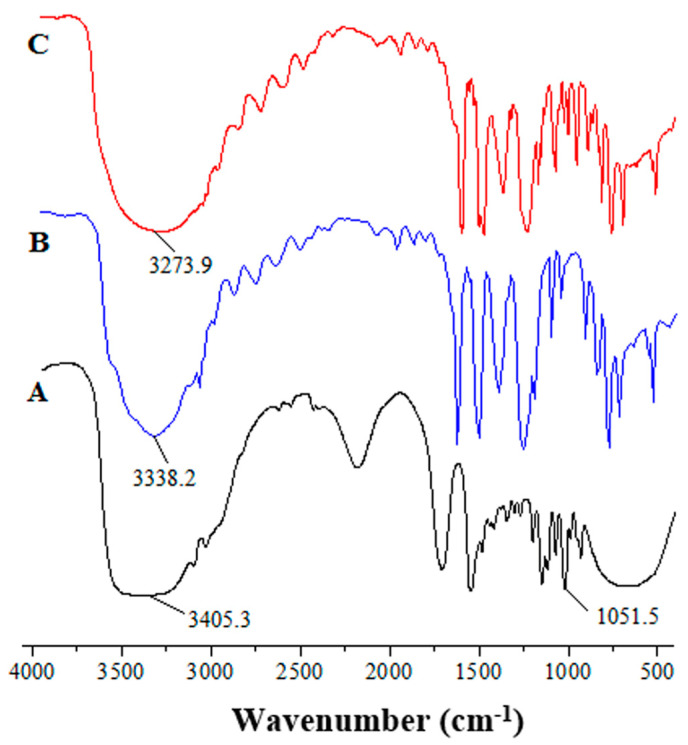
FT-IR spectra of (**A**) choline chloride (**B**) phenol and (**C**) DES.

The FT-IR spectra of choline chloride, phenol, and synthesized DES using choline chloride and phenol at a molar ratio of 1:4 were investigated. In the FT-IR spectra of phenol (Figure 1B), characteristic vibrations of O-H at 3338.2 cm^−1^ and C=C (1450–1600) cm^−1^ were observed. C-N and O-H vibrations of choline chloride were positioned at 1051.5 cm^−1^ and 3405.3 cm^−1^, respectively (Figure 1A). In the spectrum of DES of choline chloride–phenol (1:4) (Figure 1C), the stretching vibration of the hydroxyl (O-H) group shifted to 3273.9 cm^−1^, and the absorption peak of the hydroxyl group in DES was broader than that in phenol, which indicated that intermolecular hydrogen bonds were formed between choline chloride and phenol [17,25].

### 2.2. Optimization of DES-UA-DLLME Conditions

To achieve the best extraction efficiency, extraction conditions were optimized using a working sample (5 ng mL^−1^). Experimental parameters such as the type and composition of extraction solvent, type of demulsifier solvent, the volume of DES and THF, pH of the sample solution, and ultrasonic time affecting the extraction efficiency were carefully investigated.

#### 2.2.1. Single Factor Optimization

##### Effect of Type and Composition of Extraction Solvent

The choice of a suitable extraction solvent is important in DES-UA-DLLME methods. In order to fully contact the DES with the sample, the hydrophilic DES was prepared to accelerate the mass transfer of the analyte between two phases. Choline chloride (ChCl) is non-toxic, biodegradable, and inexpensive and can form DES with HBD via hydrogen bonds. The HBD usually includes carboxylic acids, urea, or polyols [26,27]. In this experiment, ethylene glycol, glycerol, and phenol mixed with ChCl to prepare DES were investigated. The experiment showed that those kinds of DESs were hydrophilic, but the DES synthesized by ethylene glycol and glycerol could not be converted into hydrophobic after adding THF, meaning that it was not easy to carry out the next phase separation operation. Therefore, DES synthesized by ChCl and phenol was selected for subsequent experiments.

The molar ratio of HBA and HBD also has a significant impact on the densities of DESs. Different molar ratios, including 1:2, 1:3, 1:4, 1:5, and 1:6 of ChCl to phenol, were examined to prepare DES-1, DES-2, DES-3, DES-4, and DES-5. The results are shown in Figure S1. It can be observed that DES-3 showed good extraction efficiency for targeted analyte. The recoveries of analytes achieved were in the range of 83.2–103.8%. So, the optimum ChCl–phenol ratio of DES was selected as 1:4 for the remaining work.

##### Effect of Type of Demulsifier Solvent

The developed method is based on the emulsification and self-aggregation of DES in aqueous solution, so the selection of a suitable demulsifier plays an important role in the effective completion of the self-aggregation and separation process. By adding an aprotic demulsifier solvent to homogenous DES aqueous phase, the interaction of water molecules with DES molecules is decreased, DES molecules could leave the water molecules and the self-aggregation process of DES molecules occurs, and an immiscible liquid is separated. The most credible and rational mechanism of DESs self-aggregation involves π-π overlap between the aromatic ring, hydrogen bonding between functional groups of DESs, and other charge transfer interactions [28]. Three kinds of demulsifier solvents, including 1,4-dioxane, THF, and dichloromethane, were investigated on the extraction efficiency. The extraction recoveries obtained with THF were much higher than 1,4-dioxane and dichloromethane. The recoveries of eight OPPs from 82.6 to 104.1% were acquired. Therefore, THF was adopted as a demulsifier solvent in the following studies.

##### Effect of pH of Sample Solution

To evaluate the sample solution pH effect on the efficiency of the method, 3.0–11.0 was investigated. The results given in Figure S2 show that the pH does not affect the recoveries obviously. However, the pH of the three samples used in this experiment was about 5.0, and satisfactory recoveries were obtained in the range of 81.6–1005.5%. So, in order to simplify the operation, the pH was not adjusted in the following experiment.

#### 2.2.2. Optimization by BBD

To optimize extraction conditions (volume of DES, volume of THF, and ultrasonic time), a 17-run BBD was applied to study the possible interaction between the parameters.

**Figure 2:**
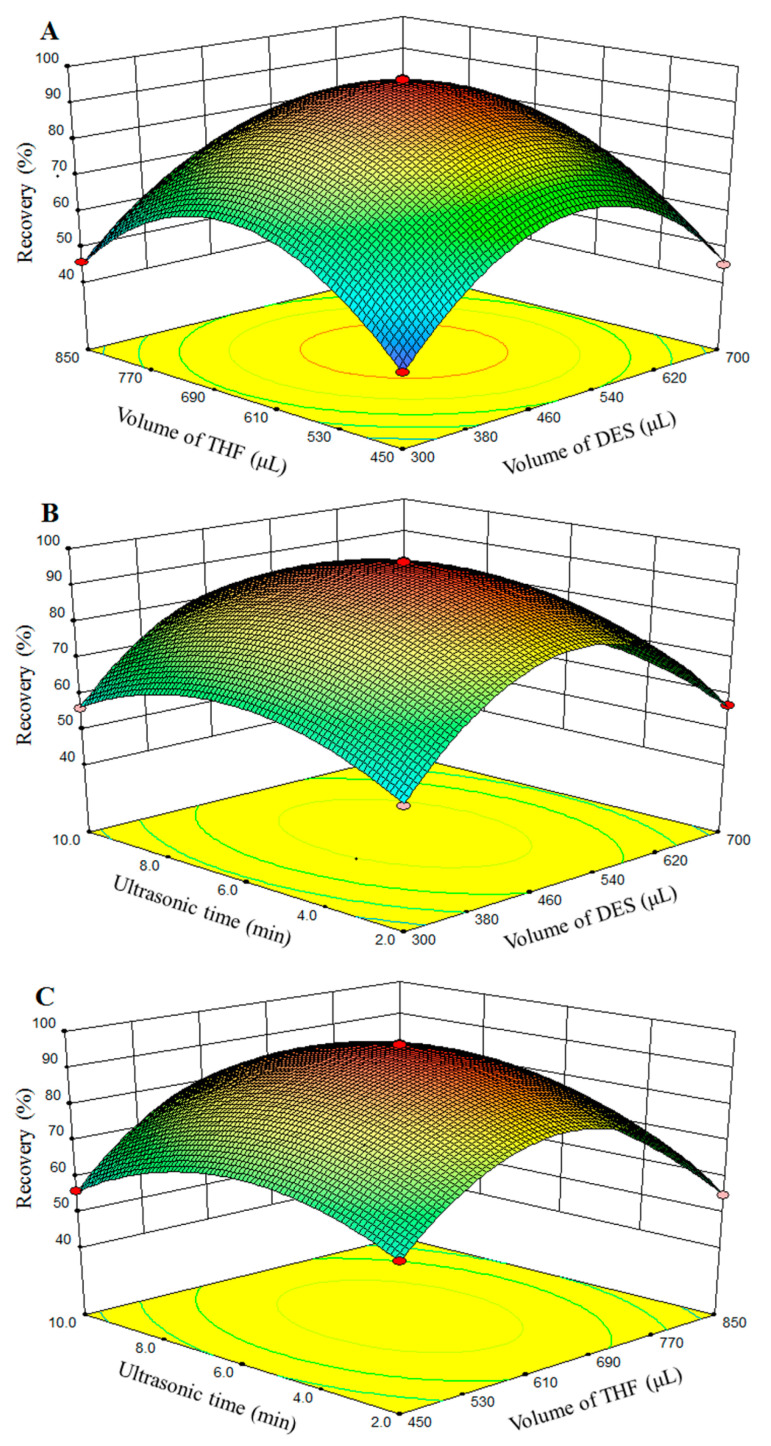
Three-dimensional response surfaces. Variables for extraction of tolclofos-methyl, (**A**) volume of THF, and volume of DES (ultrasonic time, 6 min); (**B**) ultrasonic time and volume of DES (volume of THF, 550 μL) and (**C**) ultrasonic time and volume of THF ((volume of DES, 650 μL).

The applicability of the model was evaluated by the square of the correlation coefficient (R^2^), the F-test, and the *p*-value in the analysis of variance (ANOVA). The analytical results are shown in Table 1 and Table 2. The R^2^ of Diazinon, tolclofos-methyl, pirimiphos-methly, phosalone, malathion, fenthion, fenamiphos, and bolster was 0.9995, 0.9991, 0.9982, 0.9975, 0.9993, 0.9982, 0.9984, and 0.9987, respectively, which indicated a good correlation. The *p*-value of the model was lower than 0.0001 (significant), and the lack of fit value was higher than 0.6362 (not significant), which showed that this model accurately represents the experimental data. Three response surfaces obtained in the BBD are illustrated in Figure 2. Tolclofos was selected as a representative analyte. The volume of DES and THF can affect the volume of hydrophobic DES directly. The smaller the volume of hydrophobic DES obtained, the higher the concentration of OPPs. However, quite a small volume of hydrophobic DES caused the extraction to become difficult and insufficient. Conversely, a large volume of hydrophobic DES may reduce the extraction efficiency, which can lead to the low preconcentration of the analyte in DES phase. It can be seen that the extraction recoveries of the target analytes increase with the increase in the volume of the THF increase and then reach a plateau. The main reason for this is that the hydrophobic DES phase reached its largest point. DES used as an extraction solvent interacts with target analyte molecules via strong hydrogen bonding and π-π interactions. According to the results, the volume of DES and ultrasonic time showed a significant influence on the extraction yields, and the volume of THF had only an insignificant effect on the extraction efficiencies. Finally, the optimal conditions for the extraction of OPPs were as follows: volume 650 μL of DES, 550 μL of THF, and 6 min of ultrasonic time.

### 2.3. Extraction Kinetics Studies

In order to discuss the extraction rate better, the extraction kinetics of eight OPPs by DES in different time periods were investigated. The pseudo first-order kinetic model assumes that the adsorption efficiency is controlled by diffusion. The pseudo second-order kinetic model assumes that the adsorption efficiency is determined by the square value of the number of adsorption vacancies on the adsorbent surface. The formulas of the model are as follows:

(1) $$\mathrm{lg}\left(q_{e}{-q}_{t}\right){=\mathrm{lgq}}_{e}-\frac{k_{1}t}{2.303}$$

(2) $$\frac{t}{q_{t}}=\frac{1}{k_{2}q_{e}^{2}}+\frac{t}{q_{e}}$$

q_e_ is the adsorption capacity at equilibrium (mg g^−1^) and q_t_ is the adsorption capacity (mg g^−1^) at time t. *k*_1_ and *k*_2_ are the extraction rate constants of the pseudo first-order and the pseudo second-order model, separately.

**Figure 3:**
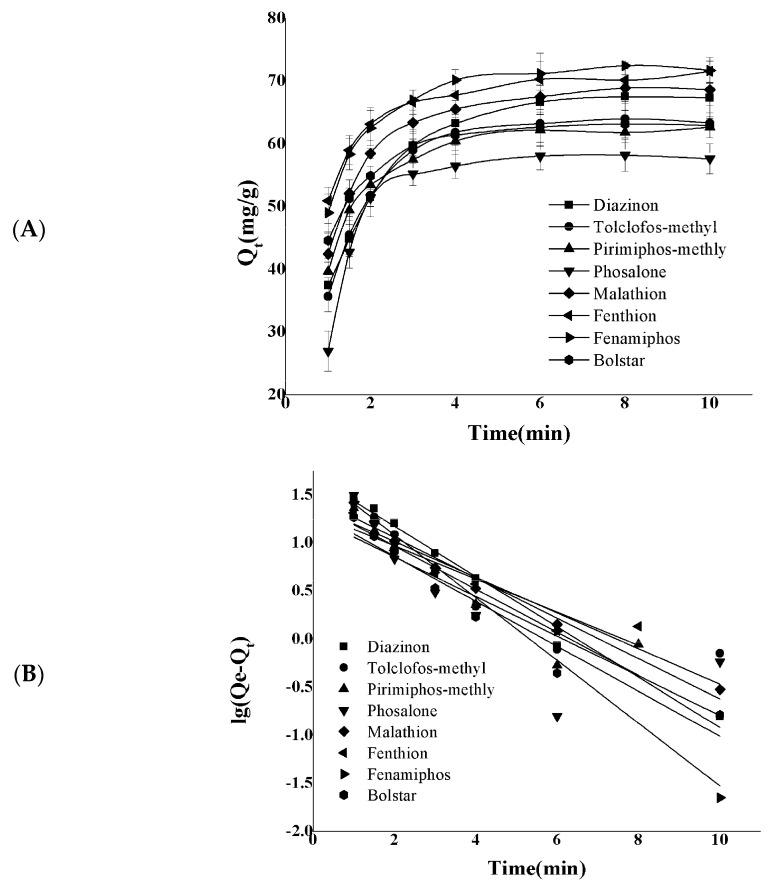

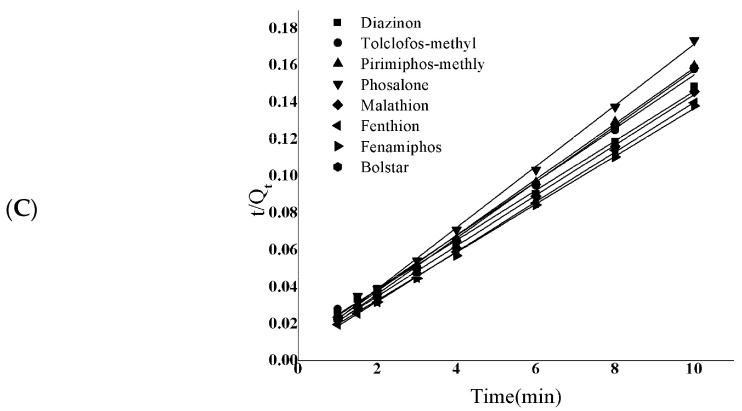
(**A**) Variation in extraction capacity of DES with time for diazinon, tolclofos-methyl, pi-imiphos-methly, phosalone, malathion, fenthion, fenamiphos, and bolster. (**B**) The pseudo first-order model, (**C**) the pseudo second-order model.

The adsorption rates of the eight analytes were fast before 2.5 min due to the large number of active sites available in the initial phase and then gradually decreased until the adsorption reached equilibrium, which was about 6 min. The kinetic data and fitting results of eight OPPs are described in Figure 3 and Table 2, and it was found that the correlation coefficient (R^2^) of the pseudo second-order kinetic model is higher than 0.991, which indicates that the main process of adsorption is chemisorption rather than diffusion.

### 2.4. Method Validation

#### 2.4.1. Analytical Performances

In order to evaluate the present method performance, the analytical characteristics of the DES-UA- DLLME procedure, including linear range, precision, the limit of detection (LOD, S/N = 3, signal-to-noise ratio), and quantification (LOQ, S/N = 10), were systematically performed. The working curves were obtained by plotting these peak areas of the analytes versus the corresponding concentrations of the analytes in the spiked honeysuckle dew samples with a wide range of 0.1–20.0 ng mL^−1^ at nine different levels (0.1, 0.2, 0.5, 1.0, 2.0, 5.0, 10.0, 15.0, and 20.0 ng mL^−1^). Good linearities were obtained with high values of the linearity and the correlation coefficient (r^2^), small values of the standard deviation of the residuals (Sy/x), the intercept (SDa), and the slope (SDb). The LODs and the LOQs of the method for actual samples are listed in Table 3. LODs and LOQs were in the range of 0.014–0.051 ng mL^−1^ and 0.045–0.170 ng mL^−1^, respectively. The experimental results demonstrated that the proposed method was appropriate for the detection of trace OPPs in honeysuckle dew samples.

#### 2.4.2. Matrix Effect

The matrix effect due to co-extracting and co-eluting matrix substances can seriously affect the analyte signals. So, the matrix effect was investigated in this work. Working curves based on the honeysuckle dew mixture sample and water were created. The matrix effect was studied and calculated by using the following equation:

$$\mathrm{Matrix}\;\mathrm{effect}\;\left(\%\right)=\frac{k_{2}}{k_{1}}\times 100$$

where *k*_2_ and *k*_1_ are the slope of the working curve based on honeysuckle dew mixture and water samples, respectively. The matrix effects are 98.3%, 93.2%, 96.7%, 97.7%, 95.1%, 98.6%, 102.6%, and 100.5% by calculation. The result shows that the working curve can perform quantitative determination, resulting in more accurate results.

#### 2.4.3. Analysis of Samples

In order to evaluate the applicability of the present method, three kinds of honeysuckle dew samples were analyzed. The typical chromatograms of the spiked sample are shown in Figure S3. No significant interference peaks were found at the retention positions of four OPPs. The spiked samples at low and high (1.0, 10.0 ng mL^−1^) degrees were analyzed to evaluate the precision and accuracy of the proposed method. The analytical results are shown in Table S1. Good recoveries of the analytes were obtained in the range of 81.4–104.4%, with relative standard deviations (RSDs) of 1.0–7.5%. In general, this present method could be satisfactorily applied for the determination of trace amounts of BAs in food samples.

#### 2.4.4. Comparison with Other Methods

The presented method based on DES-UA-DLLME was compared with other reported methods for the detection of OPPs and is listed in Table 4, including magnetic solid phase extraction (MSPE) [29], dispersive liquid–liquid microextraction (DLLME) [30], solid phase extraction (SPE) [31,32,33], and DES-DLLME [34]. It could be clearly seen that the proposed method revealed lower LODs, a wider linearity range, and satisfactory recoveries.

## 3. Materials and Methods

### 3.1. Chemicals and Reagents

Diazinon, tolclofos-methyl, pirimiphos-methly, phosalone, malathion, fenthion, fenamiphos, and bolster (≥98%) were obtained from Aladdin Chemicals (Shanghai, China), and the structures can be seen in Figure S4. Standard stock solutions for the herbicides at a concentration level of 100 μg mL^−1^ were prepared in methanol. All of the stock standard solutions were stored in a refrigerator at 4 °C. The working and mixed working standard solutions were prepared every week by diluting stock standard solutions with methanol. Analytical-reagent-grade choline chloride, phenol, glycol, glycerol, tetrahydrofuran (THF), and sodium chloride were obtained from Beijing Chemicals (Beijing, China).

### 3.2. Instrumentation

The synthesized DES was characterized by Fourier transform infrared spectroscopy (FT-IR, Nicolet FT-IR 5700, Thermo Fisher Scientific Inc., Waltham, MA, USA). A KQ3200DE ultrasonicator (Kunshan, China) was used for the sample treatment. A DELTA-320 acidity meter (Mettler-Toledo Instruments Co., Ltd., Shanghai, China) was used for the pH measurement. The phase separation was performed on an LDZ4-1.2 centrifuge (Jingli centrifuge Co. Ltd., Beijing, China).

### 3.3. Sample Preparation

Three kinds of honeysuckle dew (Samples 1–3) from different manufacturers were purchased from pharmacies (Tianjin, China). The honeysuckle dew samples were filtered. The resulting solution was shaken until it was mixed well, and then it was stored at 4 °C. The spiked honeysuckle dew samples were obtained by spiking the appropriate amount of working solution (10 μg mL^−1^) into 10 mL of sample.

### 3.4. Synthesis and Characterization of DES

**Figure 4:**
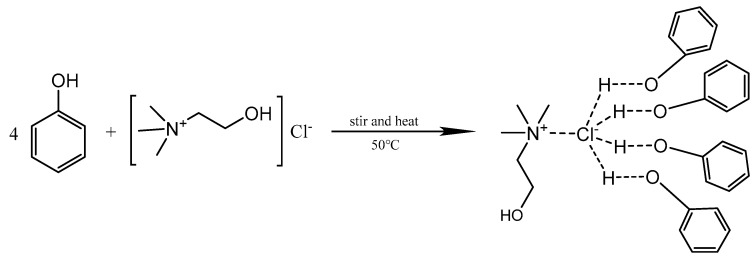
The structure of DES formation.

In this study, choline chloride and phenol were chosen to prepare DES by results reported in the previous literature [35]. In brief, choline chloride and HBD were accurately weighed, and the mixture was then heated and stirred at 50 °C until a clear liquid was obtained. During this step, the chlorine atom of choline chloride formed a hydrogen bonding with the hydrogen atom in aqueous phase, and the desired DES was formed [25]. The DES was kept in the dark at room temperature. The structure of DES formation is shown in Figure 4.

### 3.5. DES-UA-DLLME Procedure

**Figure 5:**
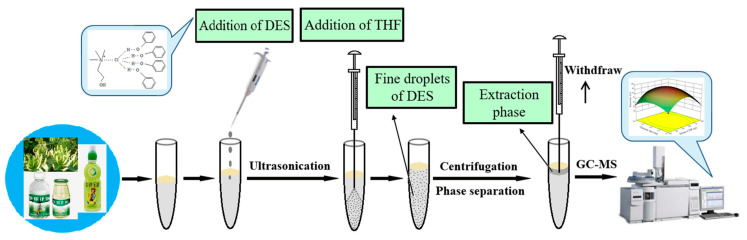
Schematic diagram of DES-UA-DLLM E system.

A schematic diagram of the DES-UA-DLLME procedure is illustrated in Figure 5. In total, 650 µL of DES as an extraction solvent was added to 10 mL of spiked honeysuckle dew sample solutions; the mixture was ultrasonicated for 6 min, and the target analytes were extracted into the extraction solvent. After extraction, 550 μL of THF was added into the homogeneous solution to aggregate the DES, and a turbid solution was formed. The solution was centrifuged at 4000 rpm for 2 min, and the upper phase was collected on the surface of the sample. Finally, an aliquot (1 μL) of the extract was injected into the GC system for analysis.

### 3.6. GC-MS Analysis

Instrumental analyses were carried out on a GCMS-QP 2010 plus (Shimadzu, Kyoto, Japan). Separations were performed on a DB-5MS capillary column (30 m × 0.25 mm I.D., film thickness of 0.25 μm, J & W Scientific, Folsom, CA, USA). The temperature program was as follows: from 70 °C to 200 °C at 15 °C min^−1^, 3 min at 200 °C, up to 250 °C at 20 °C min^−1^, 5 min at 250 °C, up to 280 °C at 25 °C min^−1^, and 2 min at 280 °C. The injection volume was 1.0 μL in the splitless mode. The mass spectrometer was operated in selective ion monitoring (SIM) mode, and the characteristic ions are given in Table 3. The injector temperature was maintained at 280 °C. The ion source and interface temperatures were 200 °C and 250 °C, respectively, and electron impact ionization energy was 70 eV.

### 3.7. Box–Behnken Design

To optimize the experimental parameters of the extraction, the volume of DES (X_1_, 350–600 μL), volume of THF (X_2_, 450–700 μL), and ultrasonic time (X_3_, 1–10 min) were employed in the Box–Behnken design (BBD) for the study. The actual design experiment is shown in Table 5. For the three factors, the equation is as follows:

$$Y=\beta_{0}+\beta_{1}X_{1}+\beta_{2}X_{2}+\beta_{3}X_{3}+\beta_{12}X_{1}X_{2}+\beta_{13}X_{1}X_{3}+\beta_{23}X_{2}X_{3}+\beta_{11}X_{1}^{2}{+\beta }_{22}X_{2}^{2}{+\beta }_{33}X_{3}^{2}$$

## 4. Conclusions

A rapid, simple, effective, and eco-friendly method of DES-UA-DLLME combined with GC-MS was successfully applied to the determination of trace amounts of OPPs in honeysuckle dew samples. The extraction, cleanup, separation, and enrichment were carried out in a single step. The smart DES was designed and synthesized as an extractant for the enrichment of trace level OPPs, which has some advantages in terms of extraction time, consumption of organic solvent, and detectability. The present method promises to have great application potential for the monitoring of pesticides at trace levels in samples.

## Figures and Tables

**Table 1:** Experimental results based on BBD.

Experiments	Coded Levels			Response: Recoveries (%)							
	X1 Volume of DES (μL)	X2 Volume of THF (μL)	X3 Ultrasonic Time (min)	Diazinon	Tolclofos-Methyl	Pirimiphos-Methyl	Phosalone	Malathion	Fenthion	Fenamphos	Bolstar
1	−1 (300)	0 (650)	1 (10)	57.1	54.7	55.3	51.4	67.7	69.4	70.4	57.3
2	0 (500)	0 (650)	0 (6)	94.1	87.4	88.2	81.3	98.2	99.9	103.2	90.3
3	−1 (300)	0 (650)	−1 (2)	53	47.2	48.8	45.6	61.1	55.6	66.6	53.8
4	0 (500)	0 (650)	0 (6)	95.1	86.2	87.3	80.2	98.3	99.3	100.3	88.5
5	−1 (300)	1 (850)	0 (6)	45.8	38.7	39.9	33.7	50.4	54.6	54.1	42.3
6	0 (500)	−1 (450)	−1 (2)	57.7	51.6	52.8	45.7	61.5	62.7	66.5	53.5
7	−1 (300)	−1 (450)	0 (6)	39.7	33.2	34.5	29.4	45.7	48.9	50.7	39.4
8	0 (500)	1 (850)	−1 (2)	55	48.6	50.1	37.5	54.6	62.3	59.3	48.7
9	0 (500)	0 (650)	0 (6)	96.2	88.2	89.7	83.2	99.5	102.6	102.1	90.9
10	0 (500)	0 (650)	0 (6)	96.5	88.9	90.1	83.2	96.8	100.5	103.5	91.8
11	0 (500)	0 (650)	0 (6)	95.2	89.2	91.8	84.5	99.2	98.5	104.6	88.5
12	1 (700)	1 (850)	0 (6)	40.9	34.7	36.3	29.3	46.3	48.5	50.8	35.4
13	1 (700)	0 (650)	−1 (2)	56.5	56.6	57.8	53.4	67.7	69.4	73	61.4
14	1 (700)	0 (650)	1 (10)	55.2	48.2	49.2	43.2	60.8	57.3	65.7	44.5
15	0 (500)	1 (850)	1 (10)	59.5	51	52.6	45.1	61.3	63.2	65.3	44.6
16	1 (700)	−1 (450)	0 (6)	45.9	40.1	41.4	33.3	49.7	53.1	54.8	41.7
17	0 (500)	−1 (450)	1 (10)	56.7	48.7	49.3	39.6	54.4	61.8	58.6	44.6

**Table 2:** Adsorption kinetics constants for eight OPPs.

Analytes	Pseudo First-Order		Pseudo Second-Order	
	k_1_ (min^−1^)	R^2^	k_2_ (g mg^−1^min^−1^)	R^2^
Diazinon	0.602	0.9862	0.019	0.9982
Tolclofos-methyl	0.427	0.8212	0.025	0.9971
Pirimiphos-methyl	0.516	0.8754	0.033	0.9991
Phosalone	0.474	0.6614	0.050	0.9994
Malathion	0.483	0.9757	0.027	0.9991
Fenthion	0.397	0.9033	0.038	0.9998
Fenamiphos	0.751	0.9812	0.028	0.9990
Bolstar	0.538	0.9413	0.043	0.9995

**Table 3:** Analytical performances.

Analytes	Retention Time (min)	Main Fragment Ion (m/z)	Regression Equation A = (a ± SDa)c + (b ± SDb)	Linear Range (ng mL^−1^)	Correlation Coefficient	LOD (ng mL^−1^)	LOQ (ng mL^−1^)	RSD n = 5 Intraday Interday	
Diazinon	15.790	304 *, 137, 152, 179	A = (848.76 ± 10.28)c − (134.75 ± 85.53)	0.2–20.0	0.9989	0.032	0.110	3.5	4.9
Tolclofos-methyl	19.094	265 *, 267, 250, 125	A = (2551.67 ± 11.20)c + (103.98 ± 21.67)	0.1–20.0	0.9999	0.016	0.051	2.7	3.7
Pirimiphos-methyl	20.878	290 *, 276, 305, 125	A = (1756.98 ± 16.90)c + (48.79 ± 13.54)	0.1–20.0	0.9994	0.015	0.048	5.5	2.9
Phosalone	22.085	182 *, 367 154,112	A = (693.12 ± 3.18)c + (42.08 ± 6.48)	0.2–20.0	0.9991	0.051	0.170	6.3	8.1
Malathion	23.209	173 *, 93, 125, 127	A = (1766.14 ± 10.36)c + (17.25 ± 5.73)	0.2–20.0	0.9991	0.014	0.045	2.6	5.3
Fenthion	27.457	278 *, 125, 109, 169	A = (1521.96 ± 5.75)c + (70.43 ± 14.80)	0.2–20.0	0.9997	0.018	0.060	2.5	4.8
Fenamiphos	29.872	303 *, 154, 80, 217	A = (1705.25 ± 3.76)c − (32.11 ± 11.23)	0.2–20.0	0.9996	0.034	0.120	4.8	4.6
Bolstar	30.611	157 *, 146, 118, 129	A = (1422.77 ± 8.00)c + (95.70 ± 6.59)	0.2–20.0	0.9992	0.023	0.076	5.2	3.8

* The ion for quantitative analysis. A, peak area of analyte; c, concentration of analyte in μg L^−1^; a, slope; b, intercept; SDa and SDb, standard deviations of slope and intercept, respectively.

**Table 4:** Comparison of some methods used for determination of OPPs.

Method	Matrix	Linear Range μg/L	LOD μg/L	Recovery (%)	RSD (%)	Ref.
MSPE	Water	100–5000	16.0–33.0	90.2–102.9	0.7–10.5	[29]
DLLME	Wine	0.2–25.0	0.025–0.88	66.7–126.1	2.0–27.2	[30]
SPE	Fruits	50.0–1000.0	10–70	88.33–120.7	1.6–3.3	[31]
SPE	Agricultural products	1–200	0.01–4.93	82.5–123.0	1.11–8.24	[32]
SPE	Water	1–50	0.002−0.118	69 to 139	0.58–8.17	[33]
DES-DLLME	Fruit juice	1–500	0.070–0.096	87.3–116.7	5.8–6.6	[34]
DES-UA-DLLME	Honeysuckle dew	0.1–20.0	0.014–0.051	81.4–104.4	1.0–8.6	this work

**Table 5:** Parameters for the BBD.

	β_0_	β_1_	β_2_	β_3_	β_12_	β_13_	β_23_	β_11_	β_22_	β_33_	*p*-Value of the Mode	Lack of Fit Value	R^2^
Diazinon	95.42	0.36	0.15	0.79	−2.78	−1.35	1.38	−27.06	−25.29	−12.91	<0.0001	0.9720	0.9995
Tolclofos-methyl	87.98	0.73	−0.075	−0.17	−2.73	−3.98	1.32	−24.80	−26.50	−11.50	<0.0001	0.9947	0.9991
Pirimiphos-methyl	89.42	0.77	0.11	−0.39	−2.62	−3.77	1.50	−24.91	−26.48	−11.74	<0.0001	0.9956	0.9982
Phosalone	82.48	−0.11	−0.30	−0.36	−2.08	−4.00	3.43	−22.32	−28.74	−11.76	<0.0001	0. 6362	0.9975
Malathion	98.40	−0.050	0.16	−0.088	−2.03	−3.38	3.45	−22.00	−28.38	−12.07	<0.0001	0.9756	0.9993
Fenthion	100.16	−0.025	0.26	0.21	−2.58	−6.48	0.45	−24.23	−24.66	−13.00	<0.0001	0.8418	0.9982
Fenamiphos	102.74	0.31	−0.14	−0.67	−1.85	−2.78	3.47	−21.82	−28.32	−11.99	<0.0001	0.9817	0.9984
Bolstar	90.00	−1.22	−1.02	−0.30	−2.30	−5.10	1.20	−21.95	−28.35	−13.80	<0.0001	0.9842	0.9987

## Data Availability

The data are contained within the article and Supplementary Materials.

## Supplementary Materials

The following supporting information can be downloaded at: https://www.mdpi.com/article/10.3390/molecules29143423/s1, Figure S1: Effect of molar ratio of choline chloride to phenol on recoveries of OPPs; Figure S2: Effect of pH of sample solution on recoveries of OPPs; Figure S3: Typical chromatograms chromatograms for the spiked sample. 1. Diazinon; 2. Tolclofos-methyl; 3. Pirimiphos-methly; 4. Phosalone; 5. Malathion; 6. Fenthion; 7. Fenamiphos; 8. Bolster; Figure S4: Structures of OPPs; Table S1: Analytical results of real samples (n = 3).
