# Supplementary material for: Deep Eutectic Solvents-Based Ultrasonic-Assisted Dispersive Liquid–Liquid Microextraction for the Determination of Organophosphorus Pesticides in Honeysuckle Dew Samples
Source: Molecules. 2024 Jul 21;29(14):3423. doi: 10.3390/molecules29143423 (PMC11279416; doi:10.3390/molecules29143423)
Supplement: Supplementary file 1 [file molecules-29-03423-s001.zip › molecules-3039076-supplementary.pdf]

Figure S1 Effect of molar ratio of choline chloride to phenol on recoveries of OPPs

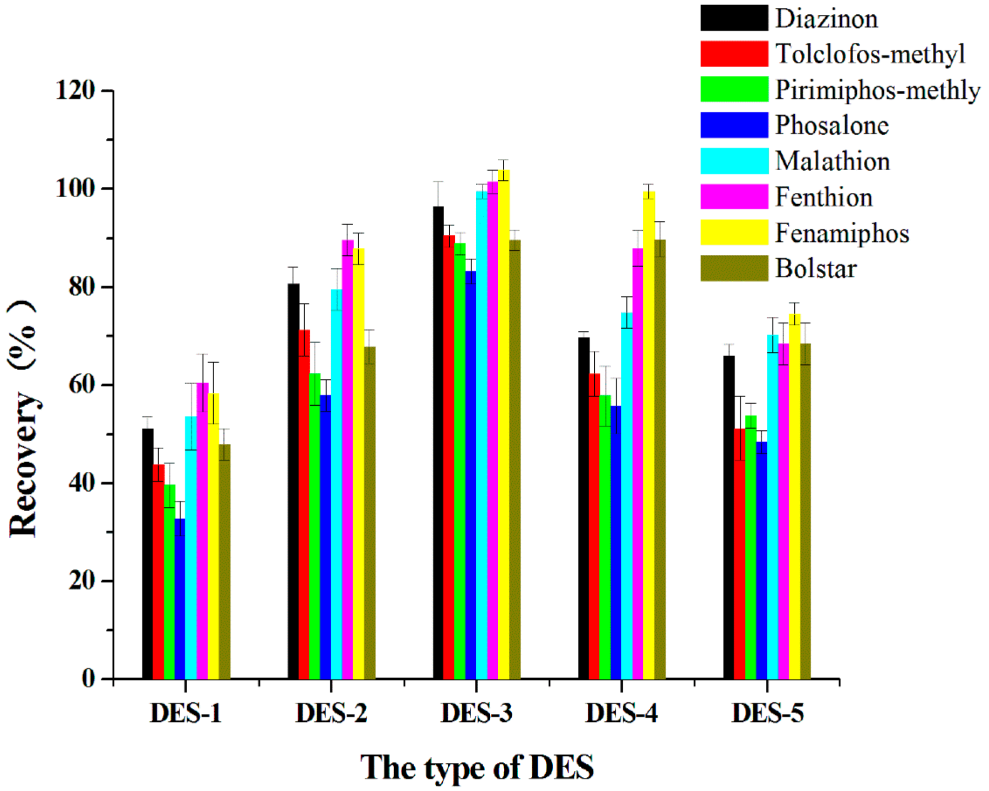

Figure S2 Effect of pH of sample solution on recoveries of OPPs

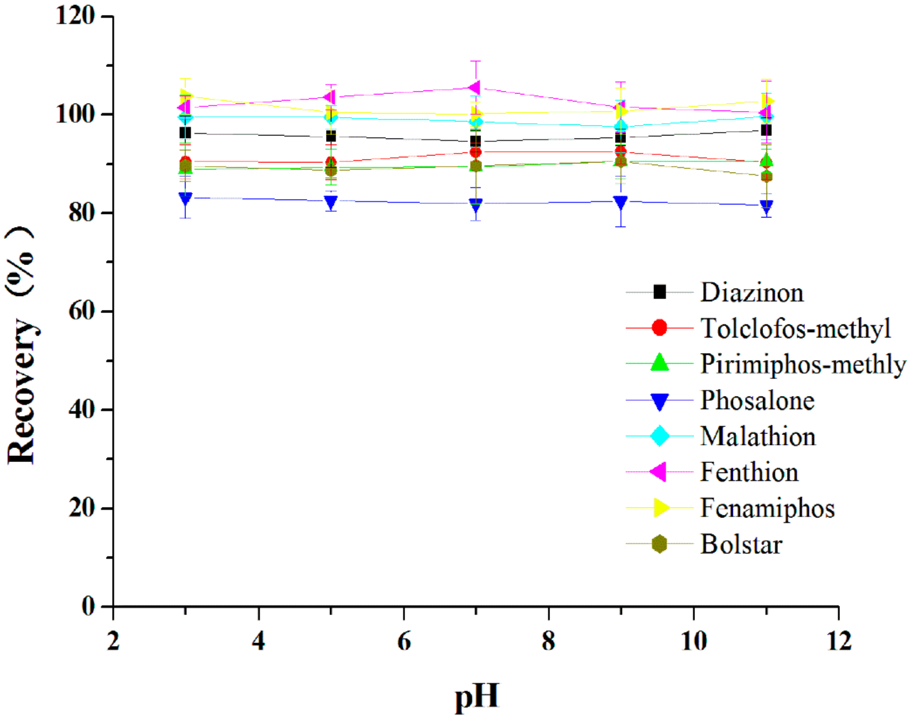

Figure S3 Typical chromatograms chromatograms for the spiked sample. 1. Diazinon; 2. Tolclofos-methyl; 3. Pirimiphos-methly; 4. Phosalone; 5. Malathion; 6. Fenthion; 7. Fenamiphos; 8. Bolster.

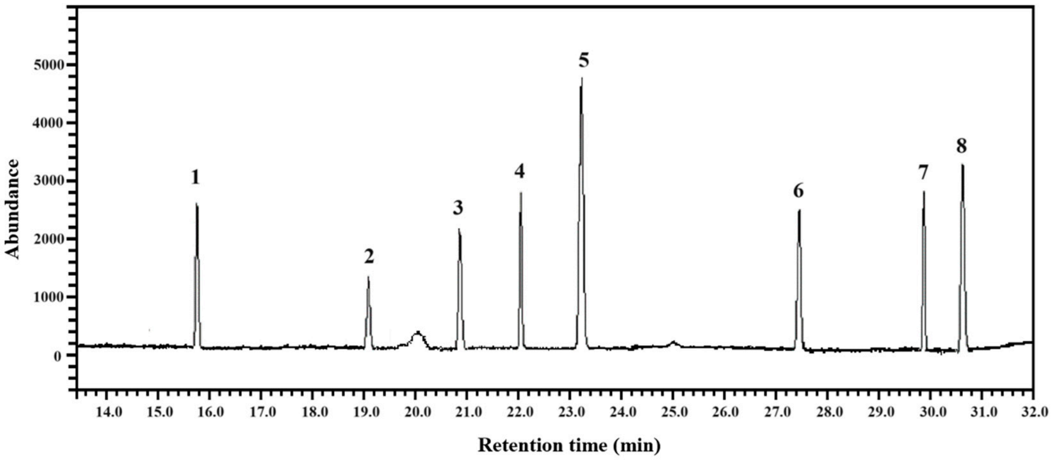

Figure S4 Structures of OPPs

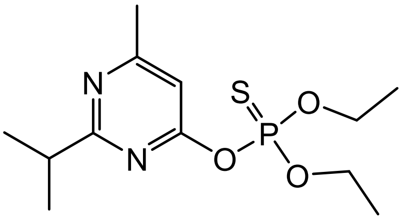

Diazinon

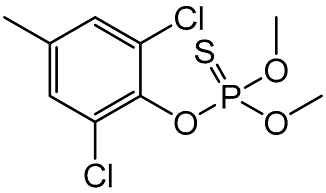

tolclofos-methyl

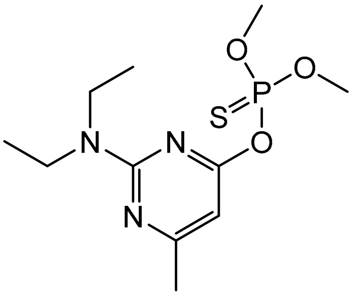

pirimiphos-methly

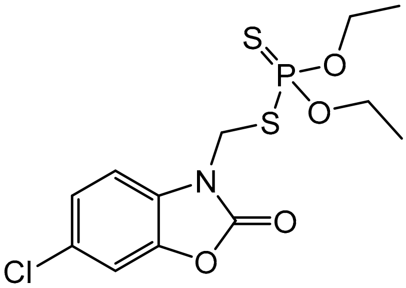

phosalone

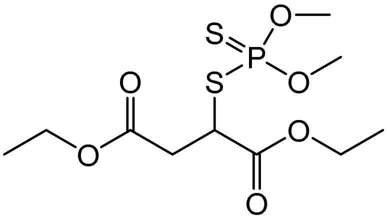

malathion

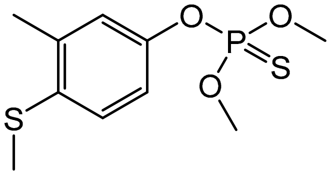

fenthion

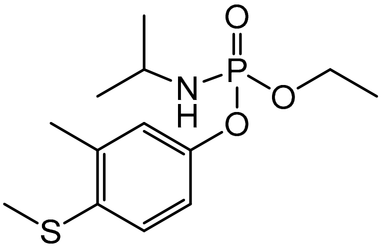

Fenamiphos

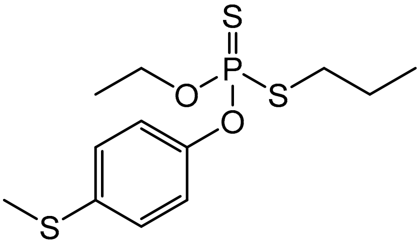

bolster

Table S1 Analytical results of real samples (n=3)

Analytes	Spiked concentration (ng mL^{-1})	Sample 1		Sample 2		Sample 3	
		Recovery (%)	RSD (%)	Recovery (%)	RSD (%)	Recovery (%)	RSD (%)
Diazinon	1.00	94.3	2.4	95.3	4.3	93.2	6.4
	10.00	95.5	3.6	93.4	2.5	91.4	4.3
Tolclofos-methyl	1.00	88.5	5.3	89.4	3.3	87.4	3.5
	10.00	90.4	3.6	89.2	4.7	84.3	3.8
Pirimiphos-methyl	1.00	86.1	7.5	86.7	2.1	88.9	2.7
	10.00	87.5	5.1	88.5	1.6	88.2	5.7
Phosalone	1.00	83.8	4.5	82.4	4.6	81.4	6.5
	10.00	82.5	4.3	85.7	6.5	83.7	8.6
Malathion	1.00	99.7	3.7	98.3	7.3	99.7	4.6
	10.00	97.9	3.8	98.7	3.9	95.6	4.8
Fenthion	1.00	103.2	5.5	101.6	1.0	100.7	3.3
	10.00	102.4	3.4	104.4	2.6	96.7	3.7
Fenamiphos	1.00	99.3	3.6	103.5	4.2	99.6	5.8
	10.00	101.5	3.9	103.1	4.8	97.8	4.2
Bolstar	1.00	89.8	6.0	90.3	3.5	90.8	4.5
	10.00	90.6	4.2	87.2	3.7	90.5	3.0
